# The Role of Collagen-Based Biomaterials in Chronic Wound Healing and Sports Medicine Applications

**DOI:** 10.3390/bioengineering8010008

**Published:** 2021-01-08

**Authors:** David A. Yeung, Natalie H. Kelly

**Affiliations:** Smith+Nephew Inc., 1450 Brooks Road, Memphis, TN 38116, USA; natalie.h.kelly@gmail.com

**Keywords:** collagen, scaffold, tissue regeneration, sports medicine, chronic wound

## Abstract

Advancements in tissue engineering have taken aim at treating tissue types that have difficulty healing naturally. In order to achieve improved healing conditions, the balance of exogenous matrix, cells, and different factors must be carefully controlled. This review seeks to explore the aspects of tissue engineering in specific tissue types treated in sports medicine and advanced wound management from the perspective of the matrix component. While the predominant material to be discussed is collagen I, it would be remiss not to mention its relation to the other contributing factors to tissue engineered healing. The main categories of materials summarized here are (1) reconstituted collagen scaffolds, (2) decellularized matrix tissue, and (3) non-decellularized tissue. These three groups are ordered by their increase in additional components beyond simply collagen.

## 1. Introduction

In the face of any injury, the ultimate goal is tissue regeneration, which restores the form and function of the native tissue as faithfully as possible. Two areas in medicine with difficulty in treating injuries include sports medicine and chronic wounds. The subset of orthopedic surgery that broadly aims to address injuries of certain tissue such as tendon, ligament, hyaline cartilage, and meniscus is often referred to as sports medicine. Similar to other tissues that are the target of engineered healing, the aforementioned types tend not to heal well naturally after injury. A major factor behind this is the lack of cellularity and vascularization present in most of these different tissues. Thus, tissue-engineered solutions are being sought after to assist in their repair. Likewise, skin wounds that do not progress through the phases of wound healing in a timely manner are considered chronic wounds and are difficult to heal. Chronic wounds are often stalled in the inflammatory phase of healing, and allograft tissues and collagenous skin substitutes aim to provide extracellular matrix (ECM), growth factors, and sometimes cellular cues to help the wound progress to healing. In particular, placental membrane allografts that are decellularized, as well as cellular allografts, have been studied extensively in the treatment of difficult-to-treat wounds such as diabetic foot ulcers (DFUs), venous leg ulcers (VLUs), and pressure injuries (PIs). Although skin substitutes have been extensively used for the treatment of hard-to-heal wounds, new interest in their use in sports medicine applications is emerging. This is due to their regenerative and healing properties including anti-inflammatory, anti-microbial, anti-fibrotic, anti-adhesion, and angiogenic properties.

The extracellular matrix itself serves an important role at all phases of wound healing. ECM proteins are important in blood clot formation, in forming a provisional matrix, and in stopping bleeding, which are all facets of the first phase of wound healing—hemostasis. During the inflammatory phase, ECM proteins stimulate cells to migrate into the wound to remove necrotic tissue and fight infection. Monocytes bind to ECM proteins, stimulating phagocytosis. Proliferation involves the binding of cells to the ECM, which stimulates cell multiplication and replication. Finally, remodeling begins with the degradation of the fibronectin ECM, which is subsequently replaced with type I collagen such that the ECM components are remodeled to provide additional strength to the damaged tissue [1]. These phases are very similar in many other soft tissues, including musculoskeletal tissues such as tendon and ligament.

In this review, we aim to describe some of the collagen-based biomaterials and allograft tissues that are currently used to augment healing in sports medicine and chronic wounds.

## 2. Collagen Scaffolds

This class of materials is typically obtained from xenograft sources. This describes the broad category of collagen-based products that have been sourced from certain collagen-rich tissue and processed. In order to achieve this commercially, it must be first purified, then packaged, and finally sterilized. In this section, the materials referred to as scaffolds are highly purified collagen that may come from any number of species or tissue types. While the source tissue may have additional physical and chemical properties, the collagen is isolated during processing. The purification typically involves disintegration, reshaping, stabilization, and drying. The shaping may be through processes such as electrospinning, shaping on to a mold, or 3D printing. Lastly, drying is often through lyophilization, which allows the scaffold to maintain a high porosity, but may also be achieved through convection or solvent drying [2]. This is a broad overview of what can be used to achieve a final product whose properties such as porosity, density, and strength can be tuned within a certain range in order to obtain a desired material. These parameters may be controlled for specific applications and may vary as the intended use changes. Of note, one key designation in this processing is the balance of additional crosslinking additives in order to allow these implants to persist for the desired time in vivo with the increased risk of foreign body response [3]. These tradeoffs in resorption time, porosity, and persistence or mechanical strength are important to consider in order to achieve the desired outcomes for the scaffold.

Collagen scaffolds have been studied in a variety of tissue injuries including tendon, ligament, cartilage, and meniscus cases. Sprague-Dawley rat models have shown bovine and porcine collagen-based scaffolds as potential treatments for Achilles repair on the basis of the final tendon strength and cellular infiltration of the scaffolds [4]. Another commonly repaired tendon is the supraspinatus of the rotator cuff. Several studies have investigated the plausibility of collagen scaffolds to aid in this repair. Histology from both sheep and human studies has shown the ability of a lyophilized, reconstituted, and lightly crosslinked collagen implant of high porosity to undergo reorganization 5 weeks after implantation to augment a rotator cuff repair [5,6]. After 3 months, additional collagen was seen on top of the implant again in both sheep and humans; at 6 months, there were no traces of the implant in the rotator cuff tissue biopsied, and new tissue resembling native tendon was in its place [5,6]. Other attempts at tendon in situ regeneration to jump start cell-scaffold interactions prior to implantation have shown supporting results. Wagenhauser et al. was able to show that tenocytes can be cultivated on a collagen sponge material as a scaffold. The initial cell viability on collagen was lower compared to a polygolic acid/polydioxanon material, but the growth rate was similar, and the final tensile strength of the collagen scaffold was higher [7]. Additionally, improved mechanical properties of collagen scaffolds after tenocyte seeding has also been observed in other studies [8]. As alluded to prior, a challenge with regeneration of tendon tissue is the inherent scarcity of native cells present [9]. Thus, some treatment strategies under development aim to expand native tendon cells in culture in vitro prior to transplantation back into the damaged host tissue. However, similar to other primary cells in culture, tendon cells have shown a propensity to shift away from their natural genetic expression profile after passaging [7,10,11]. The proper usage of correct culture conditions, stimulation, and substrate such as collagen scaffolds has been postulated to rescue or maintain the normal tenocyte genotype and phenotype. Examples of genes whose expression levels are commonly used to assess the tenocyte genotype are collagen I, collagen III, decorin, scleraxis, and tenomodulin [7,12]. Similarly, some guidelines have been suggested for implants that are aimed at repairing the rotator cuff specifically. These include the ability for cells to infiltrate, provide growth cues for such cells, be extremely biocompatible, and accommodate physiological loading [13]. These are qualities in which collagen-based scaffolds excel.

Another tissue type that bears resemblance to tendon tissue are ligaments. These tissues have also been notoriously difficult heal. An exemplary representative of this is the anterior cruciate ligament (ACL), which is a commonly torn stabilizer of the knee in young, active subjects, often requiring full reconstruction with lengthy rehabilitation [14]. Alternatives to the standard reconstruction where cadaveric or host graft tissue are used to replace the torn ACL continue to be explored. Notably, Martha Murray’s work implementing her bridge-enhanced ACL repair (BEAR) technique have shown tissue engineering advances in this area. In this technique, a collagen-based scaffold derived from bovine tissue is soaked in blood and stitched in between the tibial and femoral ends of the torn ACL [15]. A unique design feature of the collagen scaffold used in this study is its ability to absorb up to five times its weight in fluid such as blood. Functional outcomes from this procedure show potential and imaging data supports the hypothesis that the repaired ACL has more physiologically normal anatomy compared to a reconstructed graft [15,16].

One of the other canonically difficult tissues to heal is cartilage, specifically in the knee. In the case of chondral defects, a common but increasingly debated treatment is microfracture, wherein the subchondral bone is punctured in order to allow cells and growth factor-rich media to flow in to the cavity left behind by the missing cartilage [17]. In this procedure, the exposed bone underneath a cartilage defect, commonly within the knee, is perforated with small holes by a pick, awl, or drill to allow bone marrow to flow into the area that is devoid of cartilage. However, there is a growing body of literature suggesting that the material filling the defect deviates from the original hyaline cartilage and is instead fibrocartilaginous in nature [18]. Therefore, microfracture augments, which are often placed in the cartilage lesion after the subchondral bone has been repeatedly punctured, have received increased interest in terms of improving outcomes. One such addition to the microfracture regimen being explored is the collagen scaffold—even without microfracture, animal models of cartilage lesions repaired with highly porous collagen I scaffolds showed improved regeneration of cartilage tissue compared to controls [19]. In this application, a collagen scaffold that would fill the hole formed by missing cartilage would be placed on top of the bone that has been punctured by the microfracture procedure. Kwan et al. recently compared the outcomes of different cartilage repair studies where microfracture treatment was augmented with scaffolds from both animals and humans [20]. The results from these studies support that collagen scaffolds may aid the regeneration of cartilage after the bone marrow stimulation of microfracture, with one to two years of data indicating good patient outcomes with tissue that resembled native hyaline cartilage. The evidence indicates more success in smaller defects while larger ones present a more challenging scenario due, in part, to more difficulty in fixation of the scaffolds [21,22,23,24,25]. This is due to the shear and compressive forces and contact that cartilage normally experiences in joints such as the knee. Among the more interesting findings from these studies are some of the histological results showing that collagen I scaffolds used to repair cartilage defects demonstrate sufficient chondrocyte infiltration and eventual presence of significant amounts of collagen II [21]. This highlights the interplay between the initial scaffold and its importance in being an appropriate environment for cells to migrate into and allow their natural functions to take place in order to regenerate damaged tissue.

Following ligament tissue, the meniscus of the knee is a difficult-to-treat tissue that may benefit from the addition of a collagen scaffold. The menisci are two crescent-shaped pieces of tissue that stabilize the knee by forming a raised edge on top of the tibia around the perimeter [26]. They have a triangular cross-section and can be torn in a variety of ways during knee trauma. The meniscus is also unique in that it has a decreasing gradient of collagen I concentration and vascularization from exterior to interior [26,27]. It has become increasingly understood that tears of the meniscus that occur on the external and middle portions, termed the red–red and red–white zones, respectively, tend to heal more successfully than those on the inner portion (white–white zone) when repaired [28]. These results owe themselves to the vascularization in that portion of the tissue, or otherwise lack thereof. Early studies attempted to use large collagen scaffolds to replace pieces of the damaged meniscus in humans and demonstrated cellular ingrowth and remodeling of the implants. In a study following nine patients for 36 months after receiving arthroscopic surgery to implant a collagen scaffold to replace damaged meniscal tissue, these patients’ activity scores were similar to those who had undergone meniscectomy in a similar time frame. The type I collagen used for this implant was purified from bovine Achilles tendon and processed into a lyophilized sponge-like material [29]. In more difficult-to-repair tears of the meniscus, scaffold options are being explored in order to minimize the amount of meniscus tissue that must be removed, therefore preventing long-term risk for osteoarthritis. Two studies in humans have shown that encapsulating damaged meniscus tissue with a collagen scaffold during repair, particularly in complex tears that may not have been able to be treated otherwise, was able to positively impact the torn tissue. Often, these treatments are combined with further adjuncts such as platelet-rich plasma in an attempt to stimulate the site with additional growth factors [30,31].

Collagen scaffolds offer a benefit to the healing of the aforementioned tissue types because they provide a skeleton of matrix for tissue types that are low in cellularity and have difficulty regenerating on their own. The high porosity and generous cellular attachment sites of collagen in lyophilzed scaffolds allows for the infiltration of additional cells that aid in the remodeling. Biopsies of human rotator cuff tissue, when studied, showed high fibroblast infiltration into the collagen scaffold placed on top of the rotator cuff tissue at three months [5]. The lyophilized collagen I scaffolds may also present unique binding sites to cells that signal a remodeling response. The reconstituted collagen would not have the same highly ordered structure as native, healthy collagen fibrils, and thus collagen monomers would more likely be exposed. Unique membrane-bound receptors for collagen known as the discoidin domain receptors, which belong to the receptor-tyrosine kinase family, have an affinity for monomeric collagen [32,33,34]. Once bound to this ligand, these receptors have been seen to upregulate matrix metalloproteinases, and therefore may be considered as sensors of matrix microenvironment damage [35]. Therefore, collagen scaffolds used to repair these tissue types are an abundance of moderately altered matrix that initiates the remodeling process. In biopsies of rotator cuff tendon and cartilage taken six months and one year after collagen scaffold implantation, respectively, the implant material showed signs of complete resorption with new tissue having taken its place [5,36]. Much work is still being done to fully understand the mechanisms that govern the healing process of tendons, ligaments, meniscus, and cartilage, but collagen scaffolds have shown they can play a role in assisting the early stages of tissue regeneration.

Finally, wound healing poses additional challenges to healing due to the complex environment and the prevalence of infection. A unique application of collagen is noted in a study by Gottrup et al., where collagen/oxidized regenerated cellulose (ORC)/silver therapy was investigated in a randomized controlled trial to treat diabetic foot ulcers. A total of 79% of patients in the collagen/ORC/silver group were positive responders compared to 43% in the control group. Results suggest that collagen/ORC/silver normalizes the wound microenvironment and protects against infection, resulting in improved wound healing [37].

## 3. Decellularized Tissue

In addition to using collagen scaffolds that are produced through the dissolution of native tissue and reformation into matrix products, decellularized tissues have also been explored as engineered constructs. Examples of source tissue for these include tendon, dermis, placental membrane, small intestine submucosa, and cartilage. While still predominantly comprised of collagen I, an advantage these decellularized tissues have over their reconstituted counterparts is the ability to provide more mechanical support. Pridgin et al. illustrated this in human flexor tendons, wherein decellularized samples retained comparable levels of strength under uniaxial loading relative to tissue that has not been decellularized [38]. Furthermore, while the native tissue is rid of cellular components, often a large portion of embedded, latent growth factors and non-collagenous matrix proteins remain. Studies have shown that after decellularization, canine Achilles tendon and human flexor tendon tissue demonstrated similar amounts of proteoglycans and growth factors within the construct compared to native levels [38,39]. The presence of these elements may be advantageous to cellular ingrowth and incorporation of the graft into a patient’s damaged tissue. Similarly, Ruprecht et al. demonstrated that frozen, homogenized, and reconstituted meniscus tissue facilitated the repopulation of native cells into a meniscal defect. This use of tissue bridges the gap between a collagen-based scaffold alone and decellularized tissue in order to combine the strengths of each such as the porosity of a scaffold to allow cellular infiltration and the preservation of some inherent growth factors [40].

As with tendon tissue, acellular dermis has been used in different tissue reconstruction procedures and is host to many native extracellular components, some of which contribute to cellular signaling directly or through their cleavage products [41]. In the area of sports medicine, it is often used to repair massive rotator cuff tears or reconstruct the superior capsule. Dermal allografts have the benefit of maintaining a high ultimate tensile strength and being able to support tendon tissue that normally bears significant load physiologically [42,43]. Even though the composition of the tissue and orientation of the collagen in dermal tissue differs from native tendon, clinical outcomes have benefited from this type of augmentation [44]. To this end, canine studies of rotator cuff repair have shown cellular integration of dermal allografts into host tendon tissue as well as formation of new collagen adjacent to the implant at six months post-surgery. At this time point, residual dermal allograft was still present [45]. In humans, data from a biopsy four months after dermal allograft implantation for rotator cuff repair showed fibroblast infiltration beginning around the periphery of the graft [46]. However, less is known about the long-term integration and remodeling of dermal acellular allograft tissue into human tendon after repairs.

The commercial landscape of dermal allografts is complex and varied due to the many different processing strategies employed to retain or omit layers of the dermis, basement membrane, and epidermis. Therefore, skin tissue can be processed for many applications such as burns, reconstructive surgery, chronic or acute wounds, or soft tissue repair [47,48]. This review has highlighted an application of dermal allografts for sports medicine in tendon repair.

Placental tissues have been used as wound coverings for over a century [49,50], but as with any tissue, allograft shelf life and processing are important factors to facilitate clinical use. One approach is to dehydrate the tissue, leaving an intact extracellular matrix containing multiple proteins, growth factors, and cytokines with the added benefit of room temperature storage [51]. However, dehydration does not retain viable cells. Regardless, dehydrated human amnion/chorion tissue allografts (dHACM; Epifix/Amniofix, MiMedx) have been used extensively in the wound care space and have been shown to increase healing rates of chronic wounds including DFUs [52] and VLUs [53]. More recently, a patient received dHACM as an augment to hamstring autograft for ACL reconstruction with promising results including vascularization of the graft, return to normal gait at 10 weeks, and return to sport at 8 months post-operation [54]. In addition, micronized dHACM has been used in the treatment of tendon tears [55], tendinopathy [55], and plantar fasciitis [52,56]. Although not a focus of this review, micronized ECM has interesting clinical applications in musculoskeletal tissues since it is injectable and may deliver similar paracrine regenerative signals as tissue allograft [55].

Porcine small intestine submucosa (SIS; Oasis, Smith and Nephew Inc., Memphis, TN, USA) is another decellularized tissue that has a long record of clinical utilization in wound healing. SIS is an intact three-dimensional extracellular matrix that allows for host cell migration and can be stored at room temperature. Several randomized controlled trials have demonstrated the effectiveness of SIS in decreasing time to wound closure and increasing percentage of wounds that achieved closure. SIS has been studied extensively, including in the treatment of chronic ulcers [57], mixed arterial/venous and venous ulcers [58], DFUs [59,60], and pressure injuries [61,62], all demonstrating its value in wound care. The benefit of SIS in wound healing may be explained in part by in vitro studies showing that MSCs cultured on SIS had increased release of angiogenic factors compared to MSCs cultured on tissue culture plastic [63]. In addition, SIS reduces MMP activity and reduces the inhibitory effects of MMPs on keratinocyte migration [64], and supports cellular adherence, differentiation, and proliferation [65].

## 4. Non Decellularized Tissue (Cellular Tissue)

In contrast to decellularized collagen matrices, some allograft tissues are processed in a way that retains cells. One example of this is the use of placental tissues that have been preserved and processed so one or more cellular types are present. It has been shown that cryopreservation of placental tissues retains cell viability [66,67,68,69,70], but requires cold storage (vCPM, Grafix, Smith and Nephew Inc.). More recently, lyopreservation in trehalose has been developed to maintain cell viability but with the added benefit of room temperature storage (vLPM, GrafixPL, Smith and Nephew Inc.) [71,72,73,74]. The main native cells present in placental tissues include fibroblasts, epithelial cells, and mesenchymal stem cells (MSCs). MSCs serve a critical role in wound healing and regeneration due to their multilineage differentiation capacity and their ability to regulate immune and inflammatory responses [75,76,77,78]. MSCs can also potentiate regeneration through paracrine signaling by releasing biologically active molecules that affect cell migration, proliferation, and survival of surrounding cells [79,80]. In addition, placental tissues and MSCs are immune-privileged, allowing for allogeneic use [81,82].

Studies have demonstrated the regenerative properties of placental membrane allografts and have highlighted their ability to aid healing. Chronic wounds and musculoskeletal injuries are often characterized by an inflammatory environment with high levels of pro-inflammatory cytokines and proteases. Elevated and prolonged expression of inflammatory cytokines and proteases leads to the degradation of extracellular matrix and growth factors, which prevents wounds and injuries from progressing to the proliferative and remodeling phases, thus inhibiting healing [83]. Cryopreserved amniotic membrane was found to be anti-inflammatory by downregulating TNF-α and IL-1α, upregulating PGE2 and IL-10, and inhibiting collagenase [68,84]. Another bulwark to healing is the presence of infection. Placental tissues are anti-microbial, due in part to the release of antimicrobial peptides [70,85]. Fibrosis and adhesions can negatively affect wound healing and regeneration due to unchecked connective tissue formation (scarring). Placental tissues were anti-fibrotic and reduced scarring in a rabbit abdominal adhesion model [71]. Finally, many tissues require blood supply to heal and placental allografts enhance angiogenesis [66,72].

Clinical trials in chronic wounds of various etiologies have demonstrated the effectiveness of placental membrane allografts in wound healing. A retrospective review of wounds of various etiologies that had largely failed other advanced treatments had a 76% closure rate at 12 weeks [86]. Two large randomized controlled trials in DFUs and complex DFUs with exposed bone, muscle, and tendon had approximately 60% wound closure by 12–16 weeks [87,88]. A “real world” retrospective analysis of 350 wounds across 58 centers also showed 59% of patients achieved closure, even in a challenging patient cohort [89]. Finally, a prospective study examined wound closure in refractory VLUs treated with placental membrane allograft. Fifty-three percent of wounds that failed to close with standard of care went on to close with vCPM [90].

Clinical studies of placental membranes have been mostly focused on chronic wounds, but due to their regenerative properties, more interest in sports medicine applications has emerged. Sundblad et al. used viable cryopreserved umbilical tissue (vCUT) as a complementary surgical wrap in primary tendon repair in five cases. Patients experienced reduced pain and reduced transition times to functional and non-assisted ambulation in normal shoe wear as compared to historical controls managed without vCUT [91]. In addition, vCUT was used in four cases of acute Achilles tendon rupture. All patients maintained durable skin closure, had minimal scarring and edema, and were able to return to work in 8 to 10 weeks [92]. Ang et al. successfully used placental allograft in two cases of tendinopathy in the peroneal tendons and the Achilles [93]. Finally, placental membrane has been used to augment rotator cuff repair in two cases [94,95]. As mentioned previously, placental membrane is immune-privileged due to its fetal origin and cellular makeup. The main living cells are mesenchymal stem cells (MSCs), which express low levels of MHC class I antigens and lack expression of MHC class II and co-stimulatory molecules. This allows for the inhibition of activation of T lymphocytes and thus their low immunogenicity [81,82]. Lack of immunogenicity is particularly important in the context of tendon repair and other sports medicine procedures since, unlike in chronic wounds, placental tissues are implanted, and thus may interact more with vasculature and immune cells. It is important to note, however, that placental membranes do not have the stability to restore mechanical structures. Rather, they can be thought of as an augment to healing that will degrade over time.

While placental membranes can be used to help additional tissue types heal, there are some instances where a cell-retaining allograft tissue is used to replace the same type of tissue in the recipient. As discussed previously, the tissue of the meniscus poses unique challenges in terms of healing, and a growing understanding of its importance in maintaining a healthy knee joint suggests that preserving as much meniscus tissue when possible after trauma is advantageous for long-term homeostasis. Therefore, in certain cases, a meniscal allograft transplant (MAT) is appropriate. Pereira et al. lists several preservation techniques or states this tissue may exist in such as lyophilized, deep frozen, cryopreserved, or fresh [96]. Of note, this enumeration applies beyond meniscal tissue alone. The overall goal of MAT is to stave off or inhibit osteoarthritis, and to that end, studies such as that done by Verdonk et al. at 10 years after implantation show benefit to approximately 70% of the patients followed [97,98].

Another tissue-specific construct in this category is related to the hyaline cartilage of the knee. A cryopreserved, viable osteochondral allograft (CVOCA) was shown to improve the repair of cartilage in a goat model when used in conjunction with bone marrow stimulation compared to just the marrow stimulation alone [99]. This is a novel result due to the current high usage of marrow stimulation to treat cartilage defects of the knee and the desire to improve upon that technique. Geraghty et al. was able to show that the ultrastructure of hyaline cartilage was preserved in the cryopreserved implants as well as having cellularity and native proteins plus growth factors retained.

## 5. Discussion

The healing of challenging tissue types and wounds is an area that continues to advance as our understanding of the biology and new technology improves. The content discussed herein focuses largely on the matrix aspect and integration of such augments in order to address certain needs in this area. The different classifications put forth by the authors of collagen-based products—scaffolds, decellularized, and non-decellularized tissue—increase the options available to treat a myriad of tissue regeneration-related challenges across sports medicine and wound healing. While matrix-related components, such as collagen, are integral to many tissue engineering applications, it is important to note the unique needs of each tissue repair (porosity, mechanical strength, resorption rate, etc.). In addition, our overview of this topic stepped through different collagen-based materials as they increased in complexity from the pure collagen-based reconstituted scaffolds to substrates that approximate the original tissue they are created from. The aim was to illustrate how a variety of biomaterials that are primarily composed of collagen can arrive in various configurations to treat a range of pathologies and attempt to heal multiple types of tissue.

## Data Availability

Data sharing not applicable.
